# Nuclear receptor/Wnt beta-catenin interactions are regulated via differential CBP/p300 coactivator usage

**DOI:** 10.1371/journal.pone.0200714

**Published:** 2018-07-18

**Authors:** Masaya Ono, Keane K. Y. Lai, Kaijin Wu, Cu Nguyen, David P. Lin, Ramachandran Murali, Michael Kahn

**Affiliations:** 1 Department of Clinical Proteomics, National Cancer Center Research Institute, Tokyo, Japan; 2 Department of Pathology, Beckman Research Institute, City of Hope, Duarte, CA, United States of America; 3 Department of Molecular Medicine, Beckman Research Institute, City of Hope, Duarte, CA, United States of America; 4 Department of Pathology and Southern California Research Center for ALPD and Cirrhosis, University of Southern California, Los Angeles, CA, United States of America; 5 Center for Molecular Pathways and Drug Discovery, University of Southern California, Los Angeles, CA, United States of America; 6 Department of Biochemistry and Molecular Medicine, University of Southern California, Los Angeles, CA, United States of America; 7 Department of Biomedical Sciences, Research Division of Immunology, Cedars-Sinai Medical Center, Los Angeles, CA, United States of America; 8 Department of Pharmacology and Pharmaceutical Sciences, University of Southern California, Los Angeles, CA, United States of America; 9 Norris Comprehensive Cancer Center, University of Southern California, Los Angeles, CA, United States of America; Florida International University, UNITED STATES

## Abstract

Over 400 million years ago, the evolution of vertebrates gave rise to a life cycle in which the organism began to live longer particularly as an adult. To accommodate such a longer lifespan, the organism underwent adaptation, developing a mechanism for long-lived cellular homeostasis. This adaptation required a population of long-lived relatively quiescent somatic stem cells (SSCs) along with a more proliferative differentiated daughter cell population, and was necessary to safeguard the genetic attributes with which SSCs were endowed. Intriguingly, cAMP response element binding protein (CREB)-binding protein (CBP) and E1A-binding protein, 300 kDa (p300), the highly homologous Kat3 coactivators had diverged, through duplication of ancestral Kat3, immediately preceding the evolution of vertebrates, given that both CBP and p300 have been detected in nearly all vertebrates versus non-vertebrates. We now demonstrate that a relatively small, highly evolutionarily conserved, amino terminal 9 amino acid deletion in CBP versus p300, plays a critical role in allowing for both robust maintenance of genomic integrity in stem cells and the initiation of a feed-forward differentiation mechanism by tightly controlling the interaction of the nuclear receptor family with the Wnt signaling cascade in either an antagonistic or synergistic manner.

## Introduction

**Fig 1:**
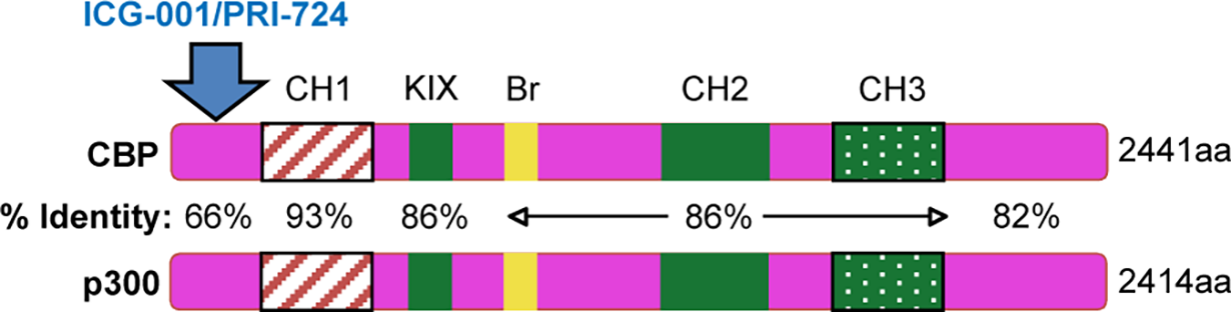
Schematic of CBP and p300 showing identity over various regions. CBP and p300 have molecular weights of approximately 300kDa and are encoded over 33 and 31 exons and consist of 2441 and 2414 amino acids (aa), respectively. β-catenin, with direct small molecule CBP/catenin (PRI-724/ICG-001) antagonist, competitively binds to CBP’s distal N-terminus, the least conserved region within these two Kat3 coactivators. CBP, cAMP response element binding protein (CREB)-binding protein; p300, E1A-binding protein, 300 kDa; Br, Bromodomain; CH, Cysteine/histidine; KIX, kinase-inducible domain interacting domain.

Over 400 million years ago, the evolution of vertebrates gave rise to a life cycle in which the organism began to live longer particularly as an adult [1]. To accommodate such a longer lifespan, the organism underwent adaptation, developing a mechanism for long-lived cellular homeostasis. This adaptation required a population of long-lived relatively quiescent somatic stem cells (SSCs) along with a more proliferative differentiated daughter cell population, and was necessary to safeguard the genetic attributes with which SSCs were endowed [1, 2]. The advent of these two different populations, a long-lived relatively quiescent population of SSCs with an “anaerobic” metabolic state inhabiting its corresponding niche and a differentiating rapidly proliferating population of daughter cells, necessitated a highly reproducible and accurate method by which to regulate cell division to be either symmetric or asymmetric [1]. Intriguingly, cAMP response element binding protein (CREB)-binding protein (CBP) and E1A-binding protein, 300 kDa (p300), the highly homologous Kat3 coactivators had diverged, through duplication of ancestral Kat3, immediately preceding the evolution of vertebrates, given that both CBP and p300 have been detected in nearly all vertebrates versus non-vertebrates [1, 3]. Although CBP and p300 diverged hundreds of millions of years ago, they are highly identical (~80%-90%) particularly in the CH1, KIX, Bromodomain, CH2 and CH3 regions (Fig 1) and even more highly similar [1, 4, 5]. These two key coactivators associate with a myriad of transcription factors to drive transcription and have previously been thought to be redundant due to being highly identical and similar proteins [1]. In fact, mounting evidence reveals that these two coactivators are indeed distinct and can execute different functions in both development and disease [1, 6–8].

**Fig 2:**
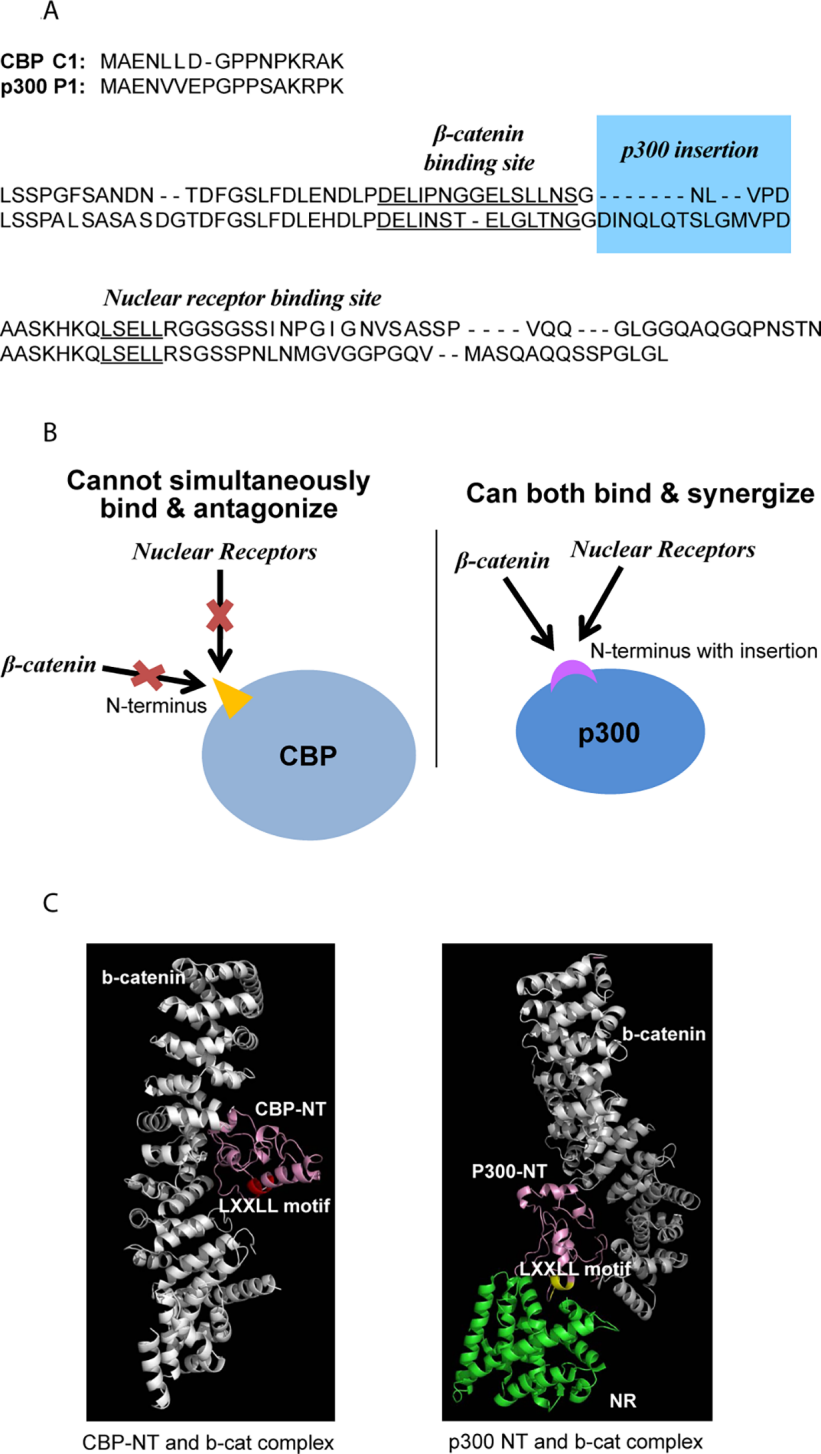
Absence of “p300 insertion” sequence sterically inhibits nuclear receptor and β-catenin from simultaneously binding to CBP; however, nuclear receptor may bind concurrently to p300 (due to lack of steric inhibition) and thereby antagonize CBP/β-catenin signaling. A. Sequence alignment of the distal N-termini of p300 (P1) and CBP (C1), depicting conserved sites for binding of β-catenin (DELI motif) and for binding of nuclear receptor (LXXLL motif), as well as the 9aa “p300 insertion” sequence which is deleted/absent from CBP. (Note: Sequences depicted are for human.) B. Absence of “p300 insertion” sequence prevents nuclear receptor and β-catenin from simultaneously binding to CBP due to steric inhibition (yellow triangle), whereas presence of the sequence allows concurrent binding and synergy of β-catenin and nuclear receptor due to abrogation of steric hindrance (lavender crescent). C. Three-dimensional structural modeling of the N-terminus of CBP/p300 and its putative interactions with β-catenin and nuclear receptor ligand binding domain of RXR alpha are shown in a ribbon representation. (Left) Interaction between the N-terminus of CBP (CBP-NT) (pink color) and β-catenin (white color) is shown. The nuclear receptor (NR) binding motif (LXXLL) is highlighted in red. (Right) Interaction between the N-terminus of p300 (p300-NT) and β-catenin (white color) and nuclear receptor (RXR alpha) (green color). The nuclear receptor binding motif in p300 (LXXLL) is highlighted in yellow color.

Within the canonical Wnt signaling cascade, β-catenin must recruit CBP or p300 in addition to other members of the basal transcription machinery to produce an active transcriptional complex [1, 9, 10]. Of interest, the respective coactivator distal N-terminus to which β-catenin and direct small molecule CBP/catenin (PRI-724/ICG-001) [1, 11, 12] or direct small molecule p300/catenin (YH 249/250) antagonists [1, 13] competitively bind, is the least conserved region within these coactivators, with only 66% identity between them (Fig 1) [1]. These distal N-termini of CBP and p300, can also recruit members of various nuclear receptor families via the LXXLL motif, which is conserved highly (Fig 2*A*) [1, 14]. However, the N-terminus within each respective coactivator is conserved highly across vertebrates; for instance, CBP of human versus mouse is 98% the same as per amino acid sequence within these N-terminal regions [1].

We previously proposed that Kat3 coactivator gene duplication generated CBP and p300 which, together with coevolution of the Wnt and nuclear receptor signaling pathways, allowed for vertebrate organisms to evolve long-term SSCs [1]. Indeed, the evolution of CBP and p300 along with Wnt/nuclear receptor signaling cascades resulted in a dependable method for maintaining the quiescent anaerobically metabolizing SSC population, which could be modulated via ligand binding to respective nuclear receptor (e.g., those involved with metabolism such as RAR, VDR, etc.) to both control asymmetric stem cell divisions and simultaneously to drive specific lineage commitment and a switch to aerobic metabolism, in order to enable development of longevity and complexity among organisms [1]. Interestingly, multiple nuclear receptor ligands including all-trans retinoic acid (ATRA) and vitamin D, along with their associated nuclear receptors, are known to counteract abnormal Wnt/β-catenin signaling, for instance in colorectal carcinoma and other cell types [15], thus phenocopying the behavior of antagonists of the CBP/β-catenin interaction by competitive binding to the N-terminal region of CBP [1]. In contrast, others have reported on the synergistic interaction of nuclear receptors and Wnts on expression of target genes [1, 16]. Intriguingly, a highly conserved and major difference in the N-terminus of CBP versus that of p300 is the 9aa deletion in the former between the DELI motif (at which β-catenin binds) and the LXXLL motif (at which nuclear receptor binds) (Fig 2*A*) [1]. Recently, we proposed a model in which such deletion furnished a mechanism by which nuclear receptors, through steric hindrance, could directly and smoothly antagonize CBP/β-catenin interaction and associated signaling, hence either maintaining the SSC quiescent state and/or effecting asymmetric divisions [1]. However, partnering with p300 (instead of CBP) has allowed for β-catenin to synergize with nuclear receptor to effect feed-forward induction of gene expression necessary for differentiation (e.g., Stra6/stimulated by retinoic acid 6) and thereby lineage specification, as steric hindrance is removed via this 9aa insertion (Fig 2*B*) [1].

Stra6 (“stimulated by retinoic acid”) belongs to a family of genes encoding transmembrane proteins and others [17]. With a Kd of 59 nM, Stra6, at the cell surface, binds with high affinity to retinol binding protein and thus is a crucial effector for cellular uptake of retinol [17]. The biological importance of Stra6 is underscored by the observation that mutations in this gene are almost always lethal perinatally [17]. Stra6 mutation results in multiple malformations / dysregulation in development which are associated with deficiency of retinoid and deletion of retinoid nuclear receptors and or proteins which play a role in metabolic pathway of retinoid [17–19]. Stra6 was initially cloned from embryonal carcinoma (P19) cells [20].

Cross-talk between canonical Wnt and RAR signaling has been previously demonstrated [21]. Treating cells with retinoid effected inhibition of a Wnt-1 reporter assay, whereas treatment with Wnt-1 effected induction of a RAR reporter assay [16]. As such, the interaction between RAR and canonical Wnt pathways may be antagonistic or synergistic. Stra6 was shown to be one of a series of genes that demonstrated significant synergistic induction by Wnt and RA in a mouse epithelial cell line [22].

To further study these dichotomous potential outcomes of Wnt and RA signaling, we utilized CRISPR/Cas9 editing to remove the 27bp insertion from p300 gene of murine embryonal carcinoma (P19) cells and now demonstrate the effect on Stra6 expression and more generally stem cell differentiation and the critical nature of this region of p300 for β-catenin’s synergistic interaction with nuclear receptor.

## Materials and methods

### Cell line

The P19 cell line (mouse embryonal carcinoma cell line) was purchased from ATCC.

### Realtime RTPCR

P19 cells were grown in MEM alpha medium containing 7.5% bovine calf serum, 2.5% fetal bovine serum, with penicillin and streptomycin. On the day prior to treatment, 200,000 cells were seeded into 6-well plates pre-coated for 1 h at room temperature with a sterile 2% gelatin solution. Cells were allowed to attach overnight before 6-h serum starvation to synchronize the cells prior to treatment in medium containing 2% serum, Wnt3a, ATRA, or a combination of Wnt3a plus ATRA. Cells were treated for 24 h and harvested with Trizol Reagent (Life Technologies). Total RNA was extracted per manufacturer’s protocol. 1 microgram of total RNA was used to carry out first strand cDNA using the cDNA Synthesis Kit (Quanta). Realtime RTPCR analysis was performed on the Bio-Rad MyiQ Single Color Real-Time PCR Detection System using PerfeCTa SYBR Green SuperMix (Quanta) and the following forward and reverse primers: *Stra6–*5`-AGCCAAGTCAGACTCCAA GAG-3’ and 5’-CAGAGAGCACACTAACTTCT TTCA-3’; *Stra8*–5’-GAGGCCCAGCATATGTC TAAC-3’ and 5’-GCTCTGGTTCCTGGTTTAAT G-3’; *Ephrin B1*–5’-GTAGGCCAGGGCTATTT CTG-3’ and 5’-TGTCTCATGAGGGTCCAAAA-3’; *Gapdh*– 5’-GGTGCTGAGTATGTCGTGGA -3’ and 5’-ACAGTCTTCTGGGTGGCAGT-3’; *Survivin–* 5’-GACAACCCGATAGAGGAGCAT -3’ and 5’-TCACTGACGGTTAGTTCTTCCA-3’.

### Immunohistochemistry/differentiation of P19 cells

P19 cells were grown in MEM alpha medium containing 7.5% bovine calf serum, 2.5% fetal bovine serum, with penicillin and streptomycin on 10cm tissue culture plates. To induce differentiation, cells were subjected to a previously described [23] protocol with minor modification. Briefly, cells were cultured for 48 h in the presence of 1μM ATRA and then dispersed with 0.25% trypsin/EDTA. The P19 cells were then seeded into fresh ATRA-containing medium and additional 24 h. Cells formed small aggregates from which individual aggregates were seeded into 6-well plates containing 20mm^2^ gelatin-coated coverslips in ATRA-containing medium. Incubation was at 37°C, 5% CO_2_ and medium was changed every two days. On day 8 (day 11 of ATRA treatment), cells were fixed and permeabilized with 4% paraformaldehyde, then stained for EphrinB1 (Santa Cruz) with secondary CF488A donkey anti-mouse IgG (Biotium), and counter-stained for DAPI (Vector Laboratories, Inc.). Immunostained cells were visualized using the Leica AF6000 Modular System fitted with a Leica CTR 6000 light/laser source and Leica DMI 6000B microscope running the Leica Application Suite Advanced Fluorescence Software. Cells were photographed under 20X magnification.

### Transient transfection

Prior to transfection, 48-well plates were coated with a 2% gelatin solution for 1 h at room temperature. P19 cells were seeded into gelatin-coated 48-well plates at 30,000 cells per well in a total volume of 200 microliters. The cells were transfected using the CalPhos Mammalian Transfection Kit (Clontech) per manufacturer’s protocol. pCDNA3.1 (empty/control vector) and pCDNA3.1-p300 wild type expression vector were provided by D. Livingston (Dana-Farber Cancer Institute, Harvard Medical School) and transfection with these plasmids was performed as previously described [24].

### Co-immunoprecipitation

Methods are as previously reported [13]. Cells were washed twice in ice-cold PBS, and then transferred into 1.5 mL eppendorf tubes chilled on ice. Cell pellets were then subjected to cytoplasmic and nuclear fractionation using the NE-PER (Pierce). Protein concentration was determined using the Bio-Rad Protein Assay. 200μg of nuclear protein from each treated sample were diluted with 1mL of co-immunoprecipitation buffer (25mM Tris, HCl, pH7.5, 150mM NaCl, 5%glycerol, 0.5%NP40, 1mM EDTA, 2mM DTT, 1X protease inhibitor cocktail). 2μg of CBP, p300, or normal rabbit IgG antibody were added to the diluted protein sample and incubated at 4°C on a rotating mixer overnight. 50μl of a 50% slurry of protein A-agarose beads, equilibrated in co-ip buffer, were added to each diluted sample containing antibody. The protein-antibody complexes were then allowed to bind to protein A-agarose on a rotating mixer for 1 h at 4°C. Protein A-agarose beads containing bound antibody/protein complex were washed three times using 500μl of co-ip buffer each time. After the final wash, the agarose beads were resuspended in 30μl of 2X Laemmli Buffer and boiled for 10 minutes for SDS-PAGE. For proteomic analysis, the agarose beads were suspended in 5% Sodium Deoxycholate (SDC) (Sigma-Aldrich). The supernatant, containing bound protein complexes were separated from the agarose beads using Illustra microspin columns. The boiled supernatants were loaded onto 8% polyacrylamide gels and subjected to SDS-PAGE. Electrophoresed proteins were then transferred onto PVDF membrane overnight under low voltage (25V) in a cold room. PVDF membranes were then probed using a mouse monoclonal antibody for β-catenin (1:5000 dilution). Detection of β-catenin was performed on the Bio-Rad ChemiDoc MP Imaging System.

### Stra6, CBP and p300 Western blotting

For detection of Stra6, P19 cells under the treated conditions were harvested and lysed using RIPA buffer (25mM Tris, pH8, 150mM NaCl, 0.1% SDS, 0.5% sodium deoxycholate and 1% TritonX-100). 50μg of whole cell lysate was subjected to SDS-PAGE and Western blotting was performed using Stra6 antibody (Santa Cruz). For CBP and p300 Western blotting, 10μg of nuclear lysate were used with antibodies to CBP and p300 (Santa Cruz).

### CRISPR/Cas9 editing system

Two guide RNAs were selected for targeting the murine Ep300 N-terminal sequence. The sequences used were: forward guide RNA 112A AGTCA GCTTCAGACAAGTCT (Chr15: 81600997–816 01016); reverse guide RNA 112B CTGACTGATATCGCCACCAT (Chr15:8160 1002–81600983). The guide RNAs were inserted into the backbone vector pCas-Guide-EF1a-GFP (Origene Technologies, Inc.). The guide RNA sequences are located within or across the boundary of the motif to be edited in murine Ep300. The vector pCas-Guide-EF1a-GFP-GE200112A (F) carrying the forward sequence AGTCAGCTTCAGACAAGTCT was chosen for the editing experiment. All sequence annotations are based on the UCSC Genome Assembly version GRCm38/mm10. The donor DNA fragment was generated by PCR using mouse genomic DNA as template and cloned into the plasmid pBluescript II sk(+) (Stratagene) to form the donor vector pBL-Donor1. The vector carries 1.8 kb of genomic DNA of murine Ep300 spanning from (Chr15:81600121) to (Chr15:81601983) (Chr15:81600991–81601023) within which the Switch motif of 33 nucleotides (Chr15:81600991–81601023) was deleted and replaced with restriction endonuclease HincII site (GTTAAC) to generate the edited sequence. For transfection of pCas-Guide-EF1a-GFP-GE200112A (F) and pBL-Donor1: P19 cells were grown in MEM alpha medium containing 7.5% bovine calf serum, 2.5% fetal bovine serum, with penicillin and streptomycin. Cells were grown on 60mm dishes. At approximately 30% confluency, the cells were transfected with pCas-Guide-EF1a-GFP-GE200112A (F) and pBL-Donor1 at 1:1 molar ratio. Transfection was performed with 8μg of DNA and 20μl of lipofectamine 2000 (Thermo Fisher Scientific).

### Colony screening

P19 cells were subcultured after 48 h following the transfection. The detached cells were suspended in growth medium and seeded into 96-well plates at a density of 30 cells per 96 wells. Noticeable single colonies formed in about 7–10 days. The colonies were subcultured in duplicate into 24-well plates at day 14 for further propagation. After nearly reaching confluency, one culture was frozen down for stock and the other prepared for PCR screening. For this purpose, cells were trypsinized, neutralized with serum-containing medium, and collected by centrifugation. The cell pellet was dissolved in 30μl lysis solution (25mM NaOH, 0.2mM EDTANa2, pH 12). The lysate was heated at 95°C for 30 min, and then neutralized with an equal volume of neutralization solution (40mM Tris-HCl, pH 5).

PCR was performed in a volume of 25μl. In each reaction, 1–2μl of cell lysate as template, 12.5μl of EmeraldAmp GT PCR Master Mix (Clontech), 0.2μM each of the forward and reverse primers were used. PCR conditions: 98°C 1 min; 98°C 10sec, 58°C 30sec, 72°C 1.5 min, 35 cycles; 72°C 5min. The PCR products were subjected to agarose gel electrophoresis. The resolved DNA bands were visualized by ethidium bromide staining and documented by photography.

The PCR primers used for screening clones and distinguishing the edited insertion region from the wild-type were specifically the forward primer detecting the edited EP300: p300-Switch-Donor3-F2 CGGCGGAGTTAACGTA CAAG (editing-generated sequence only, not present in the wild-type Ep300, located within exon 2); and the reverse primer P300-Donor-RA-R3 AGATGTTCCCAAGGCTGCTA (within intron 2–3, Chr15:81602195–81602176). The PCR product of the edited fragment was 1185bp. In the wild type primer pair, the forward primer used for detecting wild-type Ep300 was P300-Donor-LA-F3 TAAGCCTGTTCTGTCCTC AGC (within intron 1–2, Chr15: 81599715–81599735); the reverse primer specifically detecting the wild-type Ep300 was p300-Switch-R TGCCAAG ACTTGTCTGAAGCT (present in wild-type Ep300 only within exon 2, Chr15:81601021–81601001). The PCR product size for the wild-type fragment was 1307 bp.

### Sequencing genomic DNA from positive clones to confirm the results of the PCR screening

Genomic DNA samples were prepared using the Qiagen Flexigene DNA kit. The PCR enzyme PrimeStar (Clontech) was utilized. The forward primer sequence used was GGTTCACTGTTTGA CCTGGAA; the reverse primer was AGATGTT CCCAAGGCTGCTA. PCR conditions: 98°C 1min; 98°C 10sec, 58°C 15sec, 72°C 35sec, 35 cycles; 72°C 2min. The 1.3 kb PCR product generated was used as the sequencing template. The PCR products were purified from agarose gel using a Gel Extraction Kit (QIAGEN). 40ng each of DNA was sequenced by GENEWIZ. The forward sequencing primer used was GGTTCACTGTTTGACCTGGAA; the reverse sequencing primer used was GGCTGGTTC ACTGGACTGTT.

### Sequencing the cDNA edited sub-clones to define the expression ratio between different alleles

Total RNA was prepared using Trizol (Thermo Fisher Scientific) from edited clone 10. Reverse transcription of the RNA was performed with a qScript cDNA Synthesis Kit (Quanta) according to the manufacturer's protocol. PCR was performed using ClonAmp HiFi PCR Premix (Clontech) to generate a 676 bp product including the locus of edited motif. The primer sequences were forward GTTGAGTCCGCATCCCTCTC and reverse GGCTGGTTCACTGGACTGTT. PCR conditions: 98°C 10sec, 58°C 15sec, 72°C 15sec, 35 cycles; 72°C 2min. The gel-purified PCR product was inserted into the plasmid pBluescript II sk(+) and the resulting sub-clones were sequenced with T7 primer by GENEWIZ. The ratio of three modified regions in Ep300 is 1:1:1.

Clone 10: All three Switch motifs have been modified. One was precisely edited as designed; the other two were mutated by sequence specific targeting, random deletion and ligation. The size of Switch in the edited allele is 33 bp shorter than the wild type with an addition of HincII site (6 bp) as designed. The size of truncation in the two mutant alleles is 6 bp and 33 bp respectively. All three modified sequences are overlapping and the open reading frame of three Ep300 alleles remains unchanged.

### Sample preparation for proteomics

Methanol solutions of whole cell extracts were dried. To this was added 100μl of 5% SDC. Both the whole cell extracts and 100 μl of immunoprecipitates (IPs) were processed as follows. The solutions were diluted to 200μl of 1% SDC, 2M urea, 50mM NH4HCO3, 1% with 1μg trypsin and incubated at 37°C for 20 h with gentle agitation. Detailed methods are as previously reported [25] and summarized as follows. The solutions of digested peptides were treated by phase-transfer method to remove the SDC [26]. The obtained peptides were resolved with 0.1% formic acid (FA) and then quantified using a Qubit® Protein Assay Kit (Life Technologies). 3μg of peptide solution was desalted according to the manufacturer’s protocol using C18 StageTip (Thermo Scientific) [27], dried, and redissolved in 10μl of 0.1% FA. The obtained peptide solution (2μl) was subjected to nanoLC-Ultra 2D (AB SCIEX) coupled with to a TripleTOF®5600 (AB SCIEX) mass spectrometer. The subjected peptides were directly injected onto a C18 reverse-phase column (75μm×150mm; ChromXP C18-CL 3μm120 A°) (Eksigent) equipped with a nanoLC-Ultra 2D and then separated by a binary gradient consisting of mobile phase (A) 0.1% FA and 1% Acetonitrile (ACN) and mobile phase (B) 0.1% FA and 99% ACN. The gradient condition used was: 2% of B initially, increased to 25% of B at 130 min, to 90% of B at 131–135 min, and to 2% of A at 135.1 min (total run time was 155 min). The masses of the eluted peptides were determined using the TripleTOF5600.

### Proteomics analysis by 2DICAL

MS peaks were detected and quantified using 2DICAL [28, 29]. Detailed methods are as previously reported [30] and summarized as follows. 2DICAL was developed as a shotgun proteomics analysis system. It analyzes the data of mass to charge ratio (m/z), retention time (RT) and peak intensity generated by liquid chromatography and mass spectrometer (LC/MS), and each sample as elemental data; it deploys various 2-dimensional images with different combinations of axes using these four elements. From the m/z–RT image, peaks derived from the same peptide in the direction of the acquiring time are integrated. By adding algorithms to ensure reproducibility of m/z and RT, the same peak can be compared precisely across different samples, and a statistical comparison of identical peaks in different samples leads to the discovery of specific differentially expressed peptide peaks. Specific peaks are designated by their m/z and RT coordinates, and further analysis is based on these identifiers. Isotopic labeling is not necessary, and a large numbers of samples can be analyzed in this way. The peptide search engine used in 2DICAL is MASCOT® software (version 2.5.1; Matrix Science) using the Uniprot/Swiss-Prot human database (H. sapiens, 471472 sequences in Sprot_57.5 fasta file). Statistical Analyses was performed with the open-source statistical language R (version 2.7.0) [31]. The 2DICAL intensity data was converted to protein value averaging the intensity data of peptides derived from the protein.

### Three-dimensional structural modeling of the N-terminus of CBP/p300

There are no structures available for the N-terminus of CBP and p300. To understand the structural aspects of the N terminus region in CBP and p300a molecular modeling study was performed. Briefly, three-dimensional structures of the N-terminus of CBP and p300 were determined using Robetta [32]. Although the confidence levels for overall structure prediction by Robetta was low (14–16%), the secondary structure prediction was determined with higher confidence for the LXXLL motif (over 50%).

To assess whether CBP/p300 can form a complex with both β-catenin and nuclear receptor, best models, generated by the Robetta structure prediction algorithm, were used to dock with the full-length humanized β-catenin structure using β-catenin from zebrafish (PDB:2Z6G) as a template. The docking of CBP/p300 was performed using ZDOCK [33]. The top model for CBP/p300 from the ten predicted models from the ZDOCK calculations was selected for further analysis. To estimate potential binding of nuclear receptor to CBP/p300/ β-catenin complex, RXR alpha ligand binding domain (LBD) (PDB code: 1RDT) was docked to either CBP-β-catenin or p300-β-catenin complexes. In the final analysis, the docking ensembles were not optimized and only relative orientations of CBP/p300, β-catenin and RXR alpha LBD were used.

### Data analysis

Numerical data were expressed as the means ±S.E.M. unless otherwise noted. Student’s t-test or One-way ANOVA with post-hoc Tukey test was performed as appropriate. p-values < 0.05 were considered significant.

## Results

### Nuclear receptor ligand binding domain (RXR alpha) binds to p300 without any steric hindrance

De novo structure prediction by Robetta showed that the N-terminus of both CBP and p300 is highly disordered and that the nuclear receptor binding motif (LXXLL) adopted a helix. In CBP, the LXXLL motif was predicted to be part of a longer helix containing the putative β-catenin binding motif. In contrast, in p300 the LXXLL motif kept its helical structure and was solvent exposed (i.e. extended further due to the insertion of 9aa) poised for protein interaction. Further docking analyses against either β-catenin/p300 or β-catenin/CBP show that the nuclear receptor ligand binding domain (RXR alpha) binds to p300 without any steric hindrance (Fig 2C, right). On the other hand, in CBP since the LXXLL motif was part of the longer helix, the nuclear receptor did not bind (Fig 2C, left); the C-terminal of β-catenin sterically prevented both β-catenin/CBP and nuclear receptor binding.

### p300 editing affects Wnt signaling

**Fig 3:**
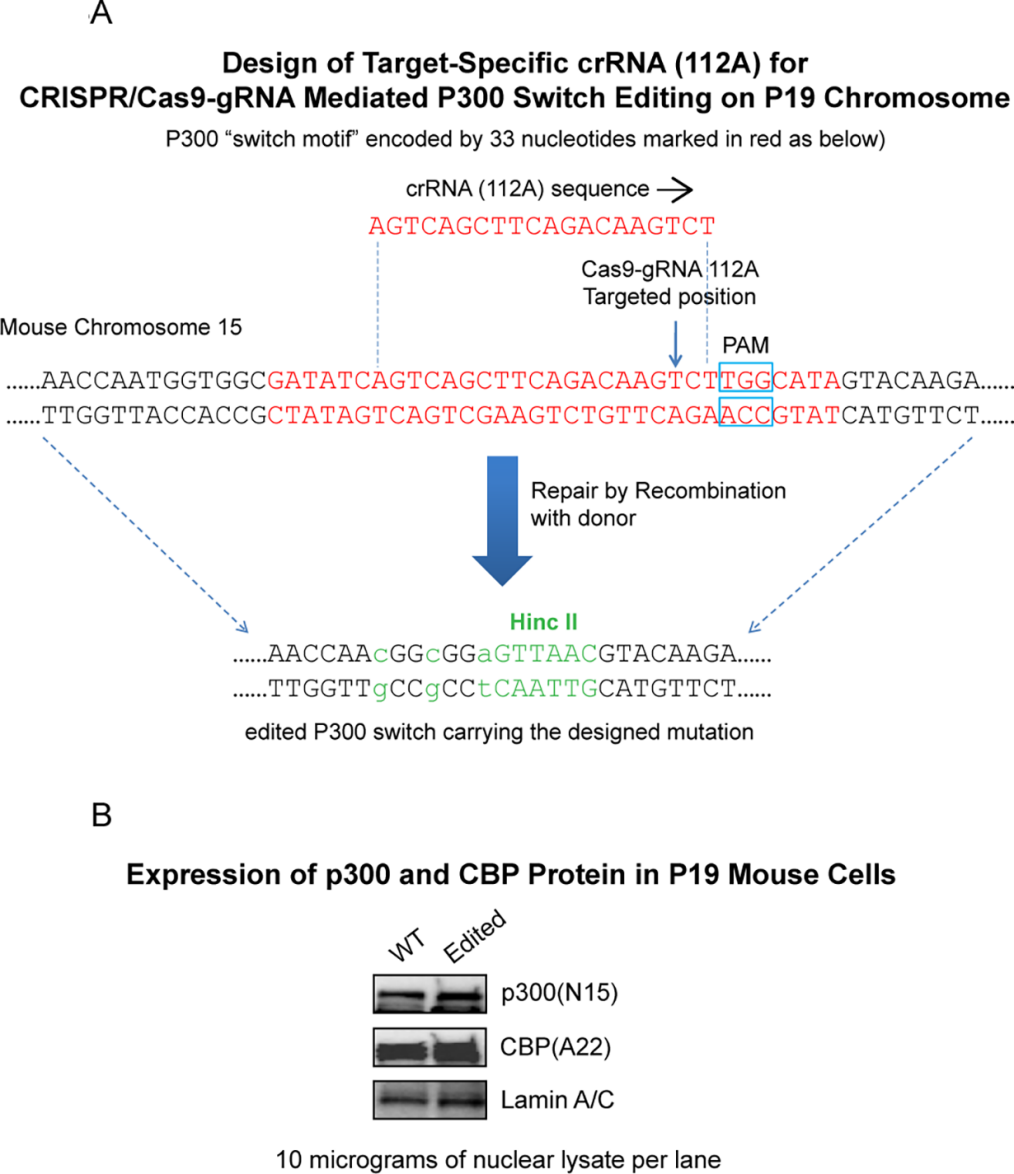
CRISPR/Cas9 editing/deletion of p300 insertion sequence in P19 mouse embryonal carcinoma cells. A. Schematic outlining the design of CRISPR/Cas9 editing in P19 mouse embryonal carcinoma cells to delete the 27bp insertion in the amino terminus of p300 (as detailed in the Experimental Procedures). Guide RNA (gRNA), CRISPR RNA (crRNA), protospacer adjacent motif (PAM). B. Immunoblotting demonstrated that protein expression levels of both CBP and p300 in the “edited P19 cells” (Edited) are equivalent to those in the wild type P19 cells (WT), confirming that the expression levels of both Kat3 coactivators were not affected by the CRISPR/Cas9 editing. n = 3.

**Fig 4:**
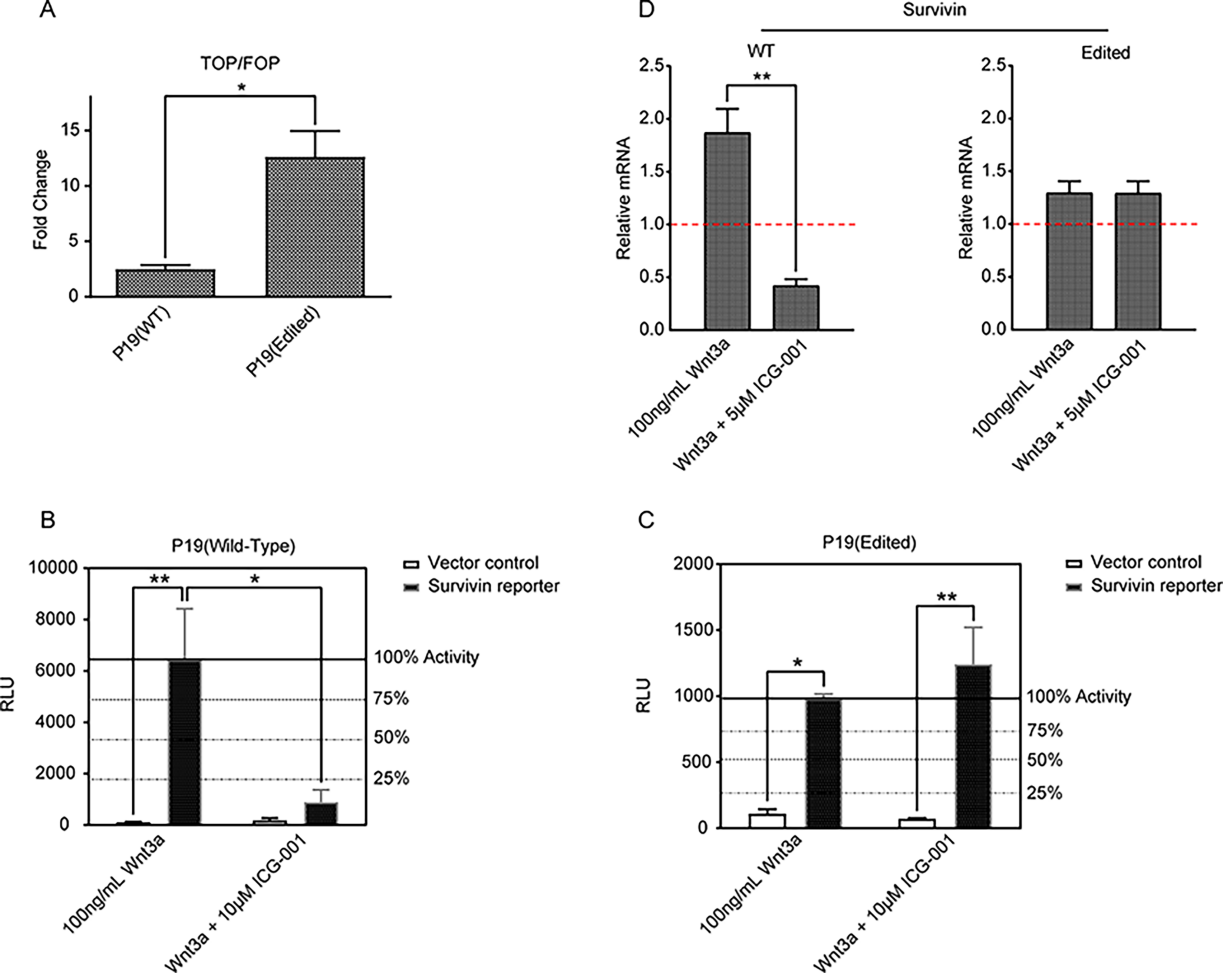
Edited P19 cells exhibited higher β-catenin/TCF transcription, as well as increased survivin/BIRC5 expression. A. Edited P19 cells exhibited significantly higher β-catenin/TCF transcription compared with wild type (WT) cells under basal conditions, as assessed by Topflash/Fopflash (TOP/FOP) activity. n = 3, *p < 0.05. B and C. In both wild type and edited P19 cells, Wnt3a significantly activated the Survivin/luciferase reporter construct (compared with empty vehicle). This activation was effectively inhibited by specific, direct small molecule CBP/catenin antagonist ICG-001 in wild type cells (B). However, in the P19 edited cells, although Wnt3a increased Survivin/luciferase activity, ICG-001 did not reduce the activity (C). Representative of n = 3, *p < 0.05, **p < 0.01. D. Expression of survivin/BIRC5 mRNA, which is highly dependent on CBP, was enhanced by Wnt3a in wild type (WT) and to a lesser extent in p300 edited P19 cells. Whereas treatment with ICG-001 in WT cells significantly decreased survivin/BIRC5 message, there was essentially no effect of ICG-001on survivin/BIRC5 expression in edited cells. n = 3, **p < 0.01. Vehicle/control data (set to 1) as indicated with a red, dashed horizontal.

We initiated our studies utilizing CRISPR/Cas9 editing in P19 mouse embryonal carcinoma cells to delete the 27bp insertion in the amino terminus of p300 (Fig 3*A*). After careful screening, we selected clone 10 in which all alleles of p300 had been edited to effectively delete the insertion (see experimental details in the Materials and Methods section). We first confirmed that in clone 10 (subsequently termed edited P19 cells) that the expression levels of both Kat3 coactivators CBP and p300 were not affected by the CRISPR/Cas9 editing. Immunoblotting (Fig 3*B*) demonstrated that the protein expression levels of both CBP and p300 in the edited P19 cells were essentially equivalent to those in the wild type P19 cells. We next tested the effects of the p300 editing on two Wnt responsive luciferase reporter constructs i.e., Topflash and Survivin/ luciferase, which we have previously utilized for first pass assessment of differential CBP/p300 Wnt/catenin coactivator usage [13]. The edited P19 cells exhibited significantly higher Topflash/Fopflash activity compared with the wild type cells under basal conditions (Fig 4*A*). In both the wild type and edited P19 cells, Wnt3a also significantly activated the Survivin/luciferase reporter construct (Fig *4B and 4C*). As previously observed [34], this activation was effectively inhibited by ICG-001 in the wild type cells (Fig 4*B*). However, in the P19 edited cells, although Wnt3a increased Survivin/luciferase activity, ICG-001 did not reduce the activity (Fig 4*C*). Furthermore, the expression of *survivin/BIRC5* message, a gene that we have previously demonstrated to be highly CBP dependent for transcriptional activation, displayed Wnt3a enhanced expression in the wild type and to a lesser extent in the p300 edited P19 cells (Fig 4*D*). Whereas treatment with ICG-001 at 5uM in the wild type cells significantly decreased *survivin/BIRC5* message, consistent with the reporter gene assay data, there was essentially no effect of ICG-001on *survivin/BIRC5* expression in the edited cells (Fig 4*D*).

**Fig 5:**
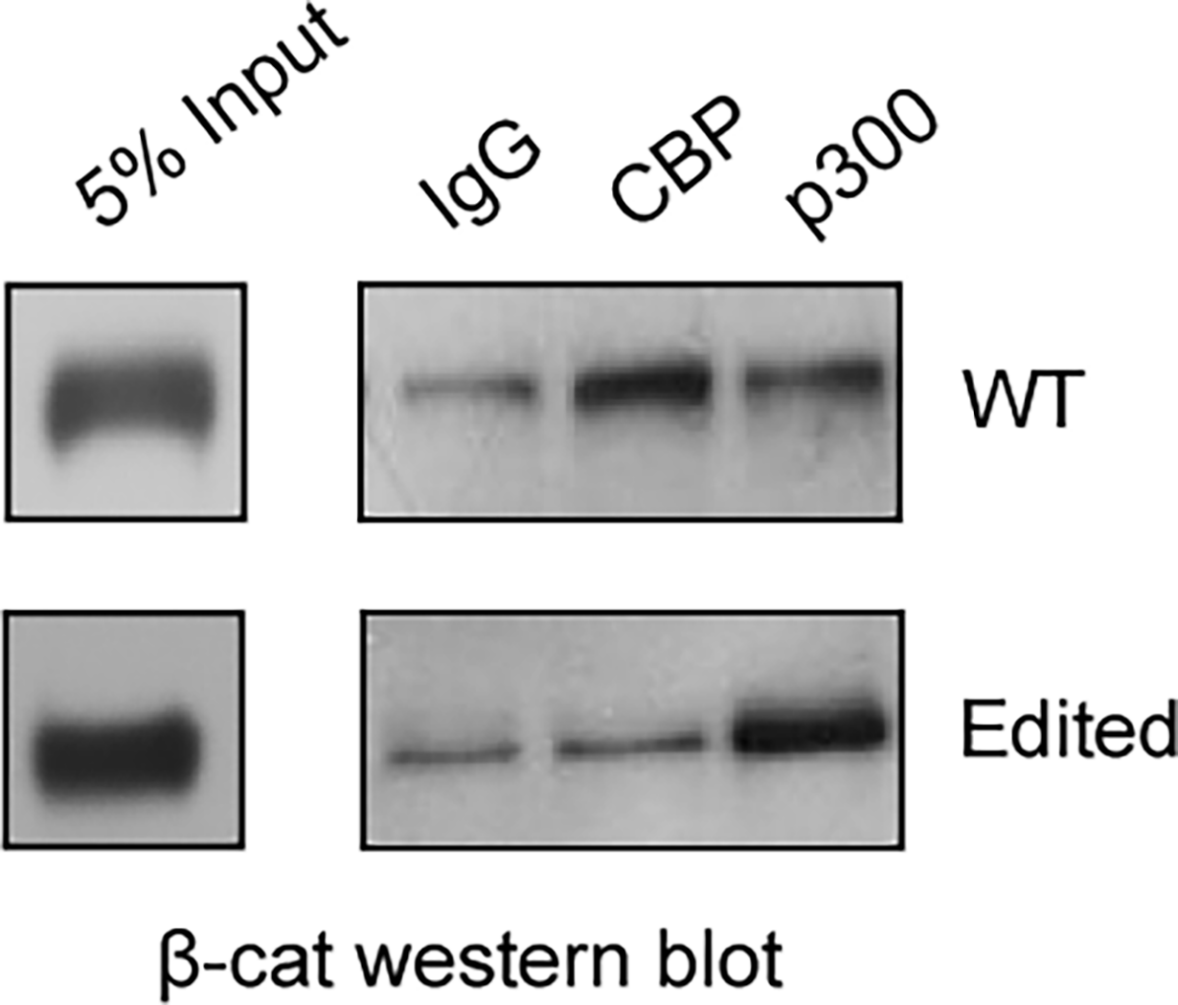
More β-catenin is associated with p300 in edited P19 cells than in wild type (WT) cells, as demonstrated by immunoblot for β-catenin using samples of nuclear protein subjected to immunoprecipitation with CBP, p300, or (control) rabbit IgG antibody. n = 3. (5% input/control immunoblotted for β-catenin is depicted on the far left).

We attribute this lack of efficacy of ICG-001 in the edited cells to the fact that although ICG-001 still binds to CBP and inhibits CBP/β-catenin transcription, it does not bind to the edited p300. (We have previously demonstrated that ICG-001 binds specifically to N-terminus of CBP but not p300 [12].) However, the edited p300, which is now more “CBP-like” due to the 9aa deletion, as opposed to wild type p300, potently drives TCF/β-catenin driven activation of both Survivin/luciferase and *survivin/BIRC5* message. To further explore differential Kat3 coactivator usage in the P19 edited cells, we used a β-catenin co-immuno-precipitation assay. β-catenin was associated with both CBP and p300 in wild type cells, but appears to be associated with only p300 in edited P19 cells (compared to IgG control). However, substantially more β-catenin appears to be associated with p300 in the edited P19 cells than in the wild type cells (Fig 5). This could potentially explain both the enhanced Topflash/Fopflash reporter activity in the edited cells, as the Topflash reporter appears to be able to utilize either Kat3 coactivator effectively and the lack of efficacy of ICG-001 in decreasing either Survivin/luciferase or *survivin/BIRC5* message [13].

### p300 editing affects Wnt and retinoic acid signaling interactions

**Fig 6:**
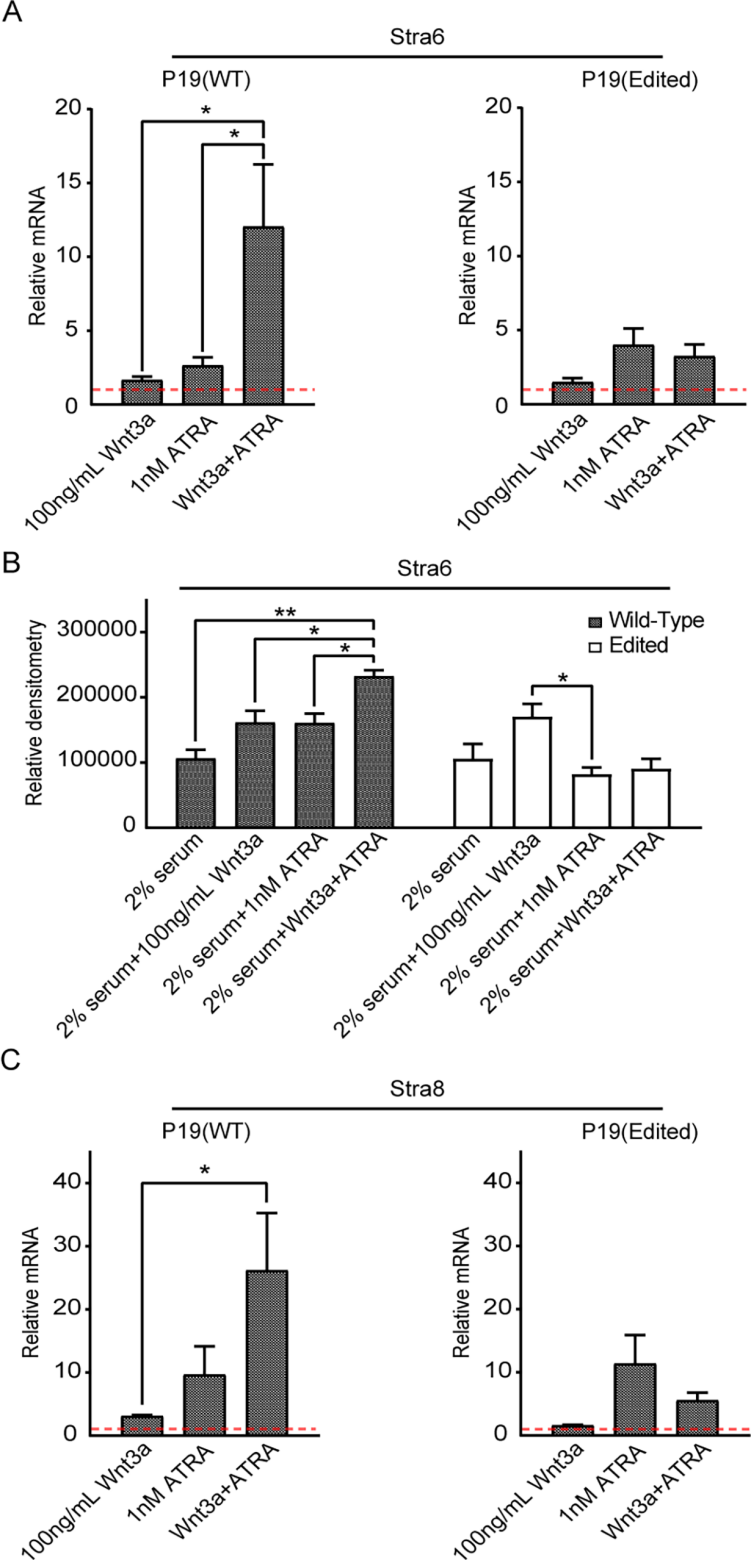
p300 editing affects Wnt and retinoic acid signaling interactions. A. Wnt3a and all-trans retinoic acid (ATRA), individually, induced Stra6 (stimulated by retinoic acid 6) mRNA expression in both wild type (WT) and p300 edited P19 cells, as assessed by realtime RTPCR. Whereas there was a strong additive effect on the expression of Stra6 with combined Wnt3a and ATRA treatment in WT cells, no additivity was demonstrated in p300 edited cells. n = 3, *p < 0.05. Vehicle/control data (set to 1) as indicated with a red, dashed horizontal. B. The additivity of Wnt3a and ATRA was also reflected at the protein level for Stra6 in wild type but not edited P19 cells, as assessed by densitometry of immunoblot for Stra6. n = 3, *p < 0.05, **p < 0.01. C. Similarly, with Stra8 (stimulated by retinoic acid 8), there is a clear additive effect of Wnt3a and ATRA in wild type (WT) cells, but there is no additive effect in edited P19 cells, as assessed by realtime RTPCR. n = 3, *p < 0.05. Vehicle/control data (set to 1) as indicated with a red, dashed horizontal.

As discussed in the introduction, ATRA via RAR/RXR has been shown to antagonize aberrant Wnt/β-catenin signaling in cancer cells [15, 21]. However, ATRA and Wnt’s synergistic induction of expression of the gene *Stra6* has also been reported [16]. We first examined the expression of *Stra6* in both wild type and p300 edited P19 cells. Although *Stra6* was expressed in both cell lines, basal expression in the p300 edited cells was 16 fold lower than in the control cells (Ct value of 28 in control versus 32 in the edited cells), consistent with the inability of the edited p300 to concomitantly bind both β-catenin and RAR/RXR to drive *Stra6* expression. Furthermore, whereas individually Wnt3a and ATRA modestly induced *Stra6* in both cell lines, there was a strong additive effect on the expression of *Stra6* with the addition of Wnt3a and ATRA in the wild type cells. However, no additivity could be demonstrated in the p300 edited cells (Fig 6*A*). Moreover, in edited P19 cells (S1 Fig, right) which have been transfected with empty/control vector (pcDNA), Wnt3a+ATRA treatment does not show any additive effect on Stra6 mRNA expression. However, wild type p300 expression vector (p300) tends to restore the additive effect (observed in WT P19 cells) of Wnt3a+ATRA on Stra6 mRNA expression in edited P19 cells. Indeed, these results further support that the 9 amino acids of p300 are important for mediating the additive effect on Stra6 mRNA expression in P19 cells. The additivity of Wnt3a and ATRA was also reflected at the protein level for Stra6 in the wild type but not the edited P19 cells (Fig 6*B*). Similarly, in the case of *Stra8 (stimulated by retinoic acid 8)*, a protein important for gametogenesis in mammals [35], although there is a clear additive effect of Wnt3a and ATRA in the wild type cells, there is no additive effect in the edited cells (Fig 6*C*).

### p300 editing may affect recruitment of SWI/SNF

To more broadly investigate the biochemical consequences of editing p300 in P19 cells, we undertook a proteomic analysis of the wild type versus the edited cells and of proteins associated with CBP and/or p300 in wild type versus p300 edited cells. One interesting feature observed from proteomic analysis of the CBP and p300 immunoprecipitation experiments was that binding to the Kat3 coactivators by SWI/SNF, a group of proteins which alter chromatin structure to facilitate transcription, was affected. SMARCD1 (BAF60A) was associated with both CBP and p300 in the wild type cells, whereas its binding to both Kat3 coactivators in the edited P19 cells was decreased (S1 Table). SMARCD1 has previously been shown to contribute to maintaining the ES cell pluripotent state [36]. The related SWI/SNF protein SMARCE1 (BAF57) in the parental P19 cells was principally associated with p300. However, in the edited P19 cells, the association of SMARCE1 to p300 was significantly reduced. This is intriguing in that the recruitment of SMARCE1 has been shown to be important for the neuronal differentiation of ES cells [37].

Moreover, many members of BAF (mammalian SWI/SNF complex) complexes, which play a role in mammalian neural development [38], including ARID1A/BAF250a, ARID2/BAF200, SMARCA4/Brg1, BCL7/BAF40, SMARCB1/hSNF5, Brd7 and SMARCC1/ BAF155 were all found associated with the Kat3a coactivators, with a number being significantly affected by the editing of p300. The heterogeneous nuclear ribonucleoprotein A2/B1 (ROA2), was found associated with both CBP and p300 in the edited cells, however, it was associated only with p300 in the parental cell line. Knockdown of ROA2 (*hnrnpa2b1*) has previously been shown to result in loss of ES cell self-renewal [39].

### Global proteomic analysis of p300 edited cells

We next performed global proteomic analysis which revealed that many proteins were also significantly differentially expressed in the P19 edited versus the parental cells. The expression of fatty acid-binding protein 5 (FABP5), which activates PPARδ, was significantly decreased (greater than 80%) in the edited cells (S2 Table). There was also a significant reduction of ALDH1A2, an enzyme that catalyzes the synthesis of ATRA from retinaldehyde in the edited cells. The mammalian ISWI chromatin remodeling protein SMARCA1 (Snf2l) was expressed only in the edited cells. Mice mutant for Snf2l display increased cellularity in the forebrain due to enhanced expansion of progenitor cells and postponed differentiation [40]. The expression level of several other interesting proteins was also dramatically down-regulated in the edited cells. For example, the expression of wdr43, a protein that is critical in ribosome biogenesis, was decreased greater than 90% in the edited cells compared with the parental cells [41], as was the SRSF8 (SFRS2B), a candidate splicing factor for the regulation of patterning and growth of the inner ear [42]. Interestingly, the expression of the histidine triad protein Hint1 was found to be significantly reduced in the p300 edited cells. Hint1 negatively regulates β-catenin/TCF-mediated transcriptional activity [43], and was increased by both Wnt3a and ATRA treatment only in the wild type P19 cells (S2 Table).

### p300 edited P19 cells do not undergo neuronal differentiation

P19 embryonal carcinoma cells have been thoroughly validated as a system for *in vitro* neuronal differentiation by ATRA [44]. *Stra6* expression, amongst a number of genes encoding proteins regulating retinoid uptake and signaling is increased upon the induction of neuronal differentiation [45]. Based upon the critical role of p300/β-catenin transcription in differentiative events [1, 10, 11, 13, 46], the lack of synergistic expression of *Stra6* by Wnt3a and ATRA and the differential core transcriptional component interactions with p300 in the edited cells, we anticipated that the p300 edited cells would exhibit neuronal differentiation defects. From the proteomic analysis, the expression of a number of proteins that are known to be important for neuronal maturation and survival, including Bcl2-associated athanogene 2 (BAG2) [47] and nardilysin (NRD1) [48] was significantly reduced in the edited cells (S2 Table). Mutations in nardilysin have previously been linked to neurodegeneration [49]. It was recently shown that NRD1 localizes to mitochondria, where it participates in folding of the enzyme α-ketoglutarate dehydrogenase, which catalyzes a rate-limiting step in the TCA cycle. Loss of Nrd1 leads to increased α-ketoglutarate, mTORC1 activation and a subsequent reduction in autophagy associated with neurodegeneration [50]. Interestingly, in this manner, p300 coactivator usage couples neuronal protection via increased autophagy to a metabolic change and increase OXPHOS [1]. Metabolic remodeling and increased OXPHOS is also a hallmark of the loss of pluripotency [51].

**Fig 7:**
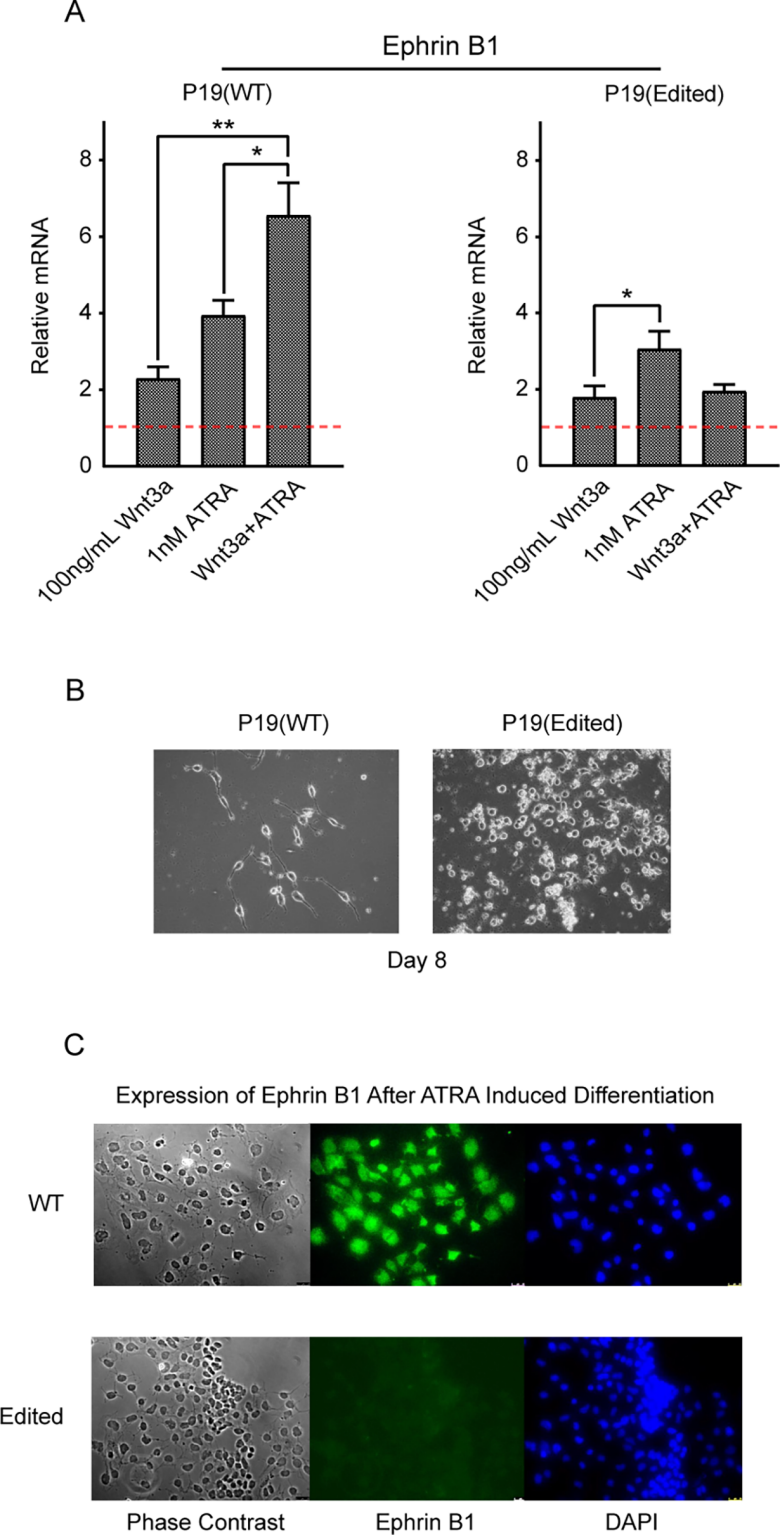
p300 edited P19 cells do not undergo neuronal differentiation. A. Expression of ephrin B1, a gene critical for neuronal migration and the maintenance of neuronal progenitors, was induced by Wnt and all-trans retinoic acid (ATRA) in an additive manner in wild type P19 cells, as assessed by realtime RTPCR. Although expression of ephrin B1 was induced by Wnt3a and ATRA, individually, there was antagonism between the two treatments in the edited cells. n = 3, *p < 0.05, **p < 0.01. Vehicle/control data (set to 1) as indicated with a red, dashed horizontal. B and C. Wild type (WT) and edited P19 cells were subjected to an established neuronal differentiation protocol (as detailed in the Experimental Procedures). As demonstrated with light microscopy, WT cells stopped proliferating and neurite outgrowth ensued, whereas edited cells continued to proliferate and neurite outgrowth was not observed (20X magnification) (B). By immuno-fluorescence microscopy, ephrin B1 was clearly expressed after subjected to the differentiation protocol in WT but not edited P19 cells (20X magnification) (C).

Similar to Stra6, ephrin B1 message expression was induced by Wnt and ATRA in an additive manner in the wild type P19 cells. Although expression of ephrin B1 was also induced by Wnt3a and ATRA, individually, in the edited cells, there was antagonism between the two treatments. (Fig 7*A*). Ephrin B1 has been demonstrated to be critical for neuronal migration and the maintenance of neuronal progenitors [52, 53] and mutations in *EFNB1* are associated with the X-linked disorder Craniofrontonasal syndrome (CFNS) (OMIM number 30411). Finally, we used a previously described neuronal differentiation protocol for P19 cells in both the wild type and edited cells. The wild type cells, as anticipated, stop proliferating and neurite outgrowth ensued, whereas the edited cells continue to proliferate and neurite outgrowth was not observed (Fig 7*B*) [44]. By both immuno-cytochemistry and proteomic analysis, ephrin B1 is clearly expressed after differentiation in the wild type but not the edited P19 cells (Fig 7*C*) (S2 Table).

## Discussion

CBP and p300 are highly identical (~80%-90%) particularly in the CH1, KIX, Bromodomain, CH2 and CH3 regions and display an even higher degree of similarity, even though they diverged more than 400 million years ago [1, 4, 5]. The distal N-termini of CBP and p300 represent the regions least conserved within these coactivators, with merely 66% identity between them [1]. Yet, each orthologous protein is highly evolutionarily conserved, approximately 98% from mouse to human [1]. CBP and p300’s distal N-terminal regions of appear to have evolved as a nexus for the integration of a number of signaling pathways (e.g., nuclear receptor family, RAR/RXR, Vitamin D, Stat1/2 and various kinase cascades) with the Wnt/β-catenin signaling cascade [1]. Both N-termini bind to β-catenin as well as to members of the nuclear receptor family via the highly conserved LXXLL motif [1, 14] and contain a myriad of serine residues as well as threonine residues which may be phosphorylated via an array of kinase cascades to control critical cellular decisions [1, 24, 54].

We previously proposed a model whereby a highly evolutionarily conserved 9 amino acid deletion in CBP between the DELI motif (where β-catenin binds) and the LXXLL motif (where nuclear receptor binds) was a critical determinant for the mode of interaction (i.e., antagonistic versus synergistic) between Wnt/β-catenin and nuclear receptor signaling [1]. To test this hypothesis, we used CRISPR/Cas9 editing in P19 embryonal carcinoma cells to remove the insertion region in p300 that lies between the N-terminal DELI sequence and the LXXLL sequence.

We demonstrated that although the expression levels of both CBP and p300 in the edited P19 cells were essentially equivalent to those in the wild type P19 cells, the edited cells exhibited significantly higher Wnt signaling as judged by the TopFlash reporter assay (Fig 4*A*). In both the wild type and edited P19 cells, Wnt3a significantly activated a Survivin/ luciferase reporter construct (Fig *4B and 4C*). This was not that surprising, as we have previously found that this reporter construct was quite “CBP-dependent” [13]. As previously observed, Survivin/luciferase and the expression of *survivin* message were effectively inhibited by ICG-001 in the wild type cells, whereas in the edited cells, ICG-001 had essentially no effect (Fig *4B, 4C* and *4D*). We attribute a compensatory effect to the edited p300 in that it is more “CBP-like” in its ability to be recruited to the promoter construct and bind β-catenin, yet it does not bind ICG-001 due to the significant primary amino acid sequence differences and therefore is not inhibited by ICG-001. As opposed to the wild type P19 cells, in the edited cells substantially more β-catenin is associated with the edited p300 (Fig 5), consistent with the enhanced Topflash reporter activity and lack of ICG-001 efficacy on Survivin/luciferase activity or *survivin/BIRC5* expression in the edited cells (Fig 4*A, 4C and 4D*).

We conclude that the edited p300 although not antagonized by ICG-001, binds β-catenin with higher avidity than wild type p300 and can thus effectively drive Wnt/β-catenin transcription in a more “CBP-like” manner, thereby having a significant impact overall on Wnt signaling.

In regards to synergy between Wnt and RAR/RXR signaling, although *Stra6* was expressed in both cell lines, basal expression in the p300 edited cells was 16 fold lower than in the control cells. As anticipated, there was a strong additive effect on the expression of *Stra6* with the addition of Wnt3a and ATRA in the wild type cells. However, no additivity could be demonstrated in the p300 edited cells for *Stra6* expression (Fig 6*A* and 6*B*). Similar results were observed for both *Stra8* and *ephrin B1* expression (Fig 6*C* and Fig 7*A*), genes that were also known to be synergistically controlled by Wnt and RAR signaling [22]. These results are fully consistent with our model outlined in Fig 2*B*.

More generally, the editing of p300 in P19 cells may affect the recruitment of the mammalian SWI/SNF complex to CBP and p300, both in regards to specific BAF recruitment, as well as distribution between the two coactivators. Specifically, SMARCD1 (BAF60A) was not significantly associated with either CBP or p300 in the edited cells, whereas it was associated with both Kat3 coactivators in the parental line. SMARCD1, which can act either as a transcriptional activator or repressor is known to be recruited by CBP/p300 to chromatin [55, 56] and contributes to the maintenance of ES cell pluripotency [36]. The heterogeneous nuclear ribonucleoprotein A2/B1 (hnrnpa2b1/ROA2) that is critical for ES cell self-renewal [39] was associated with both CBP and p300 in the edited cells; however, it was only associated with p300 in the wild type cells. We conclude that the edited P19 cells have and retain a more “stem cell-like” status. Consistent with this, the edited P19 cells failed to differentiate and produce neurites using a previously described neuronal differentiation protocol [23] and did not express the neuronal marker ephrin B1, whereas the wild type cells behaved as anticipated (Fig 7*A* and 7*B*).

The CBP/p300 association of a significant number of BAF complex members that regulate mammalian neural development including SMARCA4/Brg1, SMARCB1/hSNF5, BCL7/BAF40, Brd7 and SMARCC1/BAF155 [38] were also significantly affected by the editing of p300. Proteomic analysis demonstrated significant reduction of expression of BAG2 and NRD1 in the edited cells. Both proteins are known to play important roles in neuronal maturation and survival [47–50]. The expression of wdr43, a protein that is critical in ribosome biogenesis, was decreased greater than 90% in the edited cells compared with the parental cells [41].

Retinoid metabolism was also dramatically affected with significant decreases in ALDH1A2 protein levels. We associate the reduction in ALDH1A2 expression with the loss of a p300-dependent synergistic feed-forward ATRA induced differentiation loop, which also explains the absence in edited cells (but not in wildtype cells) of the strong additive effect on Stra6 expression resulting from combination Wnt3a and ATRA treatment.

The multiple effects on transcriptional regulation induced by the p300 editing were manifested in an obvious defect in the ability of the edited cells to differentiate. Under neuronal differentiation conditions, only the wild P19 type cells grow neurites and express the Wnt and RAR/RXR induced neuronal marker ephrin B1 [52, 53].

Multiple nuclear receptor ligands via their respective transcriptional complexes have been shown to either be antagonistic [15] or synergistic with the Wnt/β-catenin pathway [16]. We previously proposed a model to rationalize these divergent effects of nuclear receptor interactions with Wnt signaling. Simply stated, steric inhibition allows for clean nuclear receptor antagonism of CBP/β-catenin signaling, whereas utilizing p300, β-catenin and nuclear receptor pathways may synergize to effect a feed-forward mechanism to direct differentiation and lineage specification [1], as the steric constraint is removed via the highly conserved 9aa insertion (Fig 2*B*). We now have provided evidence to validate this model utilizing CRISPR/Cas9 editing of p300.

CBP/catenin antagonists and nuclear receptor ligands (e.g., ATRA, vitamin D) possess a number of commonalities both phenotypically and in effects on gene expression in stem cells. For example, acute promyelocytic leukemia is distinctly sensitive to vitamin A derivative, ATRA [1]. ATRA is not cytotoxic to the malignant leukemic cells but effects differentiation in these cells, similar to CBP/catenin antagonists [1]. Furthermore, ATRA and vitamin D, through their associated nuclear receptors, counteract abnormal Wnt signaling in colorectal carcinoma cells [1, 15], thereby mechanistically phenocopying CBP/catenin antagonists in their competitive binding to CBP’s N-terminal region.

Whereas normal levels of vitamins A and D are necessary for healthy development and are associated with healthy adulthood, abnormally elevated levels have detrimental effects, particularly while the organism is developing as evidenced by the high teratogenicity of ATRA [1]. A most impressive yet surprising finding from our previous investigations is that selective antagonism by ICG-001 of the CBP/catenin interaction, at very high concentrations, is highly safe, without any detrimental side effects, even on mouse development [46]. Mice receiving topical or oral ICG-001 at high concentrations throughout the course of pregnancy delivered normal mice, and when 6 weeks old, these progeny mice displayed normal mass and size and were capable of breeding the next generation [1]. In contrast, specifically antagonizing p300/catenin pathway in utero effects striking developmental abnormalities in nearly all organs studied [46, 57, 58]. The ligands of nuclear receptors may act as antagonists of CBP/catenin pathway; however, they are not purely CBP/catenin antagonists in that they are agonists of p300/catenin pathway [1]. Thus, whereas small molecule CBP/catenin antagonists allow for stochastic differentiation via p300, ATRA by antagonism of CBP/catenin interaction, through p300-mediated interaction, biases lineage specification and effects detrimental consequences at elevated concentrations on the developing embryo [1].

The closest orthologs of the Kat3 co-activators (CBP and p300) in other non-vertebrate species have a much extended “spacer” sequence situated in between the motifs for β-catenin binding and nuclear receptors binding than either human CBP or p300 [1]. We had previously proposed that: 1) Ancestral Kat3 coactivators are able to bind β-catenin, together with nuclear receptors or without them, to induce symmetrical or asymmetrical division of the stem cell division with the potential for opposing or cooperative interaction across the two distinct binding regions [1]; 2) CBP evolution in vertebrate organisms such that CBP binding with nuclear receptor antagonizes β-catenin/CBP interaction, has been a major breakthrough which enabled a robust method for regulating both quiescence and asymmetric divisions in SSCs of the longer-lived vertebrate organism (versus shorter-lived organism which did not require such a method/mechanism) [1]. In contrast, vertebrate p300 provided a transcriptional coactivator enabling lineage commitment and diversity. This Kat3 coactivator gene duplication and “division of labor” thereby generated a robust asymmetric/symmetric switch to tightly regulate long-lived SSCs in vertebrates. We had proposed that this increased fidelity was critical for maintaining the genetic attributes of SSCs for the increased complexity and longevity of vertebrate species [1].

In summary, we have demonstrated in the current study that the amino terminal relatively small 9 amino acid deletion in CBP versus p300, which has been highly evolutionarily conserved in vertebrates, appears to play a critical role in allowing for both robust maintenance of genomic integrity in SSCs as well as initiating feed-forward differentiation mechanism by tightly controlling the interactions between nuclear receptors and the Wnt pathway in either an antagonistic or synergistic manner. Further analysis of the differential roles of the N-termini of CBP and p300 are underway and to be reported in due course.

## Supporting information

S1 Fig p300 editing affects Wnt and retinoic acid signaling interactions. (PDF)

S1 Table 2DICAL data for IP experiment. (PDF)

S2 Table 2DICAL data for whole cell experiment. (PDF)

## References

[1] Thomas PD, Kahn M. Kat3 coactivators in somatic stem cells and cancer stem cells: biological roles, evolution, and pharmacologic manipulation. Cell Biol Toxicol. 2016;32(1):61–81. Epub 2016/03/23. 10.1007/s10565-016-9318-0 . 27008332 PMC7458431

[2] Trosko JE, Kang KS. Evolution of energy metabolism, stem cells and cancer stem cells: how the warburg and barker hypotheses might be linked. Int J Stem Cells. 2012;5(1):39–56. ; PubMed Central PMCID: PMCPMC3840988. 24298354 10.15283/ijsc.2012.5.1.39 PMC3840988

[3] Mi H, Muruganujan A, Thomas PD. PANTHER in 2013: modeling the evolution of gene function, and other gene attributes, in the context of phylogenetic trees. Nucleic Acids Res. 2013;41(Database issue):D377–86. Epub 2012/11/27. 10.1093/nar/gks1118 ; PubMed Central PMCID: PMCPMC3531194. 23193289 PMC3531194

[4] Arany Z, Sellers WR, Livingston DM, Eckner R. E1A-associated p300 and CREB-associated CBP belong to a conserved family of coactivators. Cell. 1994;77(6):799–800. . 8004670 10.1016/0092-8674(94)90127-9

[5] Eckner R, Ewen ME, Newsome D, Gerdes M, DeCaprio JA, Lawrence JB, et al Molecular cloning and functional analysis of the adenovirus E1A-associated 300-kD protein (p300) reveals a protein with properties of a transcriptional adaptor. Genes Dev. 1994;8(8):869–84. . 7523245 10.1101/gad.8.8.869

[6] Kung AL, Rebel VI, Bronson RT, Ch'ng LE, Sieff CA, Livingston DM, et al Gene dose-dependent control of hematopoiesis and hematologic tumor suppression by CBP. Genes Dev. 2000;14(3):272–7. ; PubMed Central PMCID: PMCPMC316359. 10673499 PMC316359

[7] Yamauchi T, Oike Y, Kamon J, Waki H, Komeda K, Tsuchida A, et al Increased insulin sensitivity despite lipodystrophy in Crebbp heterozygous mice. Nat Genet. 2002;30(2):221–6. Epub 2002/01/30. 10.1038/ng829 . 11818964

[8] Roth JF, Shikama N, Henzen C, Desbaillets I, Lutz W, Marino S, et al Differential role of p300 and CBP acetyltransferase during myogenesis: p300 acts upstream of MyoD and Myf5. EMBO J. 2003;22(19):5186–96. 10.1093/emboj/cdg473 ; PubMed Central PMCID: PMCPMC204457. 14517256 PMC204457

[9] Moon RT. Wnt/beta-catenin pathway. Sci STKE. 2005;2005(271):cm1 Epub 2005/02/15. 10.1126/stke.2712005cm1 . 15713948

[10] Teo JL, Kahn M. The Wnt signaling pathway in cellular proliferation and differentiation: A tale of two coactivators. Adv Drug Deliv Rev. 2010;62(12):1149–55. Epub 2010/10/21. 10.1016/j.addr.2010.09.012 . 20920541

[11] McMillan M, Kahn M. Investigating Wnt signaling: a chemogenomic safari. Drug Discov Today. 2005;10(21):1467–74. 10.1016/S1359-6446(05)03613-5 . 16243267

[12] Emami KH, Nguyen C, Ma H, Kim DH, Jeong KW, Eguchi M, et al A small molecule inhibitor of beta-catenin/CREB-binding protein transcription [corrected]. Proc Natl Acad Sci U S A. 2004;101(34):12682–7. 10.1073/pnas.0404875101 ; PubMed Central PMCID: PMCPMC515116. 15314234 PMC515116

[13] Higuchi Y, Nguyen C, Yasuda SY, McMillan M, Hasegawa K, Kahn M. Specific Direct Small Molecule p300/β-Catenin Antagonists Maintain Stem Cell Potency. Curr Mol Pharmacol. 2016;9(3):272–9. . 26008738 10.2174/1874467208666150526155146

[14] Heery DM, Kalkhoven E, Hoare S, Parker MG. A signature motif in transcriptional co-activators mediates binding to nuclear receptors. Nature. 1997;387(6634):733–6. 10.1038/42750 . 9192902

[15] Dillard AC, Lane MA. Retinol decreases beta-catenin protein levels in retinoic acid-resistant colon cancer cell lines. Mol Carcinog. 2007;46(4):315–29. 10.1002/mc.20280 . 17219422

[16] Szeto W, Jiang W, Tice DA, Rubinfeld B, Hollingshead PG, Fong SE, et al Overexpression of the retinoic acid-responsive gene Stra6 in human cancers and its synergistic induction by Wnt-1 and retinoic acid. Cancer Res. 2001;61(10):4197–205. . 11358845

[17] Blaner WS. STRA6, a cell-surface receptor for retinol-binding protein: the plot thickens. Cell Metab. 2007;5(3):164–6. 10.1016/j.cmet.2007.02.006 . 17339024

[18] Metzler MA, Sandell LL. Enzymatic Metabolism of Vitamin A in Developing Vertebrate Embryos. Nutrients. 2016;8(12). Epub 2016/12/15. 10.3390/nu8120812 ; PubMed Central PMCID: PMCPMC5188467. 27983671 PMC5188467

[19] Chambon P. The nuclear receptor superfamily: a personal retrospect on the first two decades. Mol Endocrinol. 2005;19(6):1418–28. 10.1210/me.2005-0125 . 15914711

[20] Bouillet P, Oulad-Abdelghani M, Vicaire S, Garnier JM, Schuhbaur B, Dollé P, et al Efficient cloning of cDNAs of retinoic acid-responsive genes in P19 embryonal carcinoma cells and characterization of a novel mouse gene, Stra1 (mouse LERK-2/Eplg2). Dev Biol. 1995;170(2):420–33. 10.1006/dbio.1995.1226 . 7649373

[21] Easwaran V, Pishvaian M, Salimuddin, Byers S. Cross-regulation of beta-catenin-LEF/TCF and retinoid signaling pathways. Curr Biol. 1999;9(23):1415–8. . 10607566 10.1016/s0960-9822(00)80088-3

[22] Tice DA, Szeto W, Soloviev I, Rubinfeld B, Fong SE, Dugger DL, et al Synergistic induction of tumor antigens by Wnt-1 signaling and retinoic acid revealed by gene expression profiling. J Biol Chem. 2002;277(16):14329–35. Epub 2002/02/06. 10.1074/jbc.M200334200 . 11832495

[23] Martins AH, Resende RR, Majumder P, Faria M, Casarini DE, Tárnok A, et al Neuronal differentiation of P19 embryonal carcinoma cells modulates kinin B2 receptor gene expression and function. J Biol Chem. 2005;280(20):19576–86. Epub 2005/03/14. 10.1074/jbc.M502513200 . 15767251

[24] Rieger ME, Zhou B, Solomon N, Sunohara M, Li C, Nguyen C, et al p300/β-Catenin Interactions Regulate Adult Progenitor Cell Differentiation Downstream of WNT5a/Protein Kinase C (PKC). J Biol Chem. 2016;291(12):6569–82. Epub 2016/02/01. 10.1074/jbc.M115.706416 ; PubMed Central PMCID: PMCPMC4813575. 26833564 PMC4813575

[25] Kamita M, Mori T, Sakai Y, Ito S, Gomi M, Miyamoto Y, et al Proteomic analysis of ligamentum flavum from patients with lumbar spinal stenosis. Proteomics. 2015;15(9):1622–30. Epub 2015/03/18. 10.1002/pmic.201400442 . 25641790

[26] Masuda T, Tomita M, Ishihama Y. Phase transfer surfactant-aided trypsin digestion for membrane proteome analysis. J Proteome Res. 2008;7(2):731–40. 10.1021/pr700658q . 18183947

[27] Rappsilber J, Ishihama Y, Mann M. Stop and go extraction tips for matrix-assisted laser desorption/ionization, nanoelectrospray, and LC/MS sample pretreatment in proteomics. Anal Chem. 2003;75(3):663–70. . 12585499 10.1021/ac026117i

[28] Ono M, Shitashige M, Honda K, Isobe T, Kuwabara H, Matsuzuki H, et al Label-free quantitative proteomics using large peptide data sets generated by nanoflow liquid chromatography and mass spectrometry. Mol Cell Proteomics. 2006;5(7):1338–47. Epub 2006/03/21. 10.1074/mcp.T500039-MCP200 . 16552026

[29] Ono M, Matsubara J, Honda K, Sakuma T, Hashiguchi T, Nose H, et al Prolyl 4-hydroxylation of alpha-fibrinogen: a novel protein modification revealed by plasma proteomics. J Biol Chem. 2009;284(42):29041–9. Epub 2009/08/20. 10.1074/jbc.M109.041749 ; PubMed Central PMCID: PMCPMC2781450. 19696023 PMC2781450

[30] Ono M, Kamita M, Murakoshi Y, Matsubara J, Honda K, Miho B, et al Biomarker Discovery of Pancreatic and Gastrointestinal Cancer by 2DICAL: 2-Dimensional Image-Converted Analysis of Liquid Chromatography and Mass Spectrometry. Int J Proteomics. 2012;2012:897412 Epub 2012/07/10. 10.1155/2012/897412 ; PubMed Central PMCID: PMCPMC3400370. 22844596 PMC3400370

[31] Matsubara J, Ono M, Negishi A, Ueno H, Okusaka T, Furuse J, et al Identification of a predictive biomarker for hematologic toxicities of gemcitabine. J Clin Oncol. 2009;27(13):2261–8. Epub 2009/03/16. 10.1200/JCO.2008.19.9745 . 19289617

[32] Kim DE, Chivian D, Baker D. Protein structure prediction and analysis using the Robetta server. Nucleic Acids Res. 2004;32(Web Server issue):W526–31. 10.1093/nar/gkh468 ; PubMed Central PMCID: PMCPMC441606. 15215442 PMC441606

[33] Pierce BG, Wiehe K, Hwang H, Kim BH, Vreven T, Weng Z. ZDOCK server: interactive docking prediction of protein-protein complexes and symmetric multimers. Bioinformatics. 2014;30(12):1771–3. Epub 2014/02/14. 10.1093/bioinformatics/btu097 ; PubMed Central PMCID: PMCPMC4058926. 24532726 PMC4058926

[34] Ma H, Nguyen C, Lee KS, Kahn M. Differential roles for the coactivators CBP and p300 on TCF/beta-catenin-mediated survivin gene expression. Oncogene. 2005;24(22):3619–31. 10.1038/sj.onc.1208433 . 15782138

[35] Tedesco M, La Sala G, Barbagallo F, De Felici M, Farini D. STRA8 shuttles between nucleus and cytoplasm and displays transcriptional activity. J Biol Chem. 2009;284(51):35781–93. 10.1074/jbc.M109.056481 ; PubMed Central PMCID: PMCPMC2791008. 19805549 PMC2791008

[36] Hong F, Fang F, He X, Cao X, Chipperfield H, Xie D, et al Dissecting early differentially expressed genes in a mixture of differentiating embryonic stem cells. PLoS Comput Biol. 2009;5(12):e1000607 Epub 2009/12/18. 10.1371/journal.pcbi.1000607 ; PubMed Central PMCID: PMCPMC2784941. 20019792 PMC2784941

[37] Pirotte D, Wislet-Gendebien S, Cloes JM, Rogister B. Neuregulin-1 modulates the differentiation of neural stem cells in vitro through an interaction with the Swi/Snf complex. Mol Cell Neurosci. 2010;43(1):72–80. Epub 2009/09/23. 10.1016/j.mcn.2009.09.003 . 19781646

[38] Son EY, Crabtree GR. The role of BAF (mSWI/SNF) complexes in mammalian neural development. Am J Med Genet C Semin Med Genet. 2014;166C(3):333–49. Epub 2014/09/05. 10.1002/ajmg.c.31416 ; PubMed Central PMCID: PMCPMC4405377. 25195934 PMC4405377

[39] Magdeldin S, Yamamoto T, Tooyama I, Abdelalim EM. New proteomic insights on the role of NPR-A in regulating self-renewal of embryonic stem cells. Stem Cell Rev. 2014;10(4):561–72. 10.1007/s12015-014-9517-0 . 24798243

[40] Yip DJ, Corcoran CP, Alvarez-Saavedra M, DeMaria A, Rennick S, Mears AJ, et al Snf2l regulates Foxg1-dependent progenitor cell expansion in the developing brain. Dev Cell. 2012;22(4):871–8. 10.1016/j.devcel.2012.01.020 ; PubMed Central PMCID: PMCPMC4580287. 22516202 PMC4580287

[41] Zhao C, Andreeva V, Gibert Y, LaBonty M, Lattanzi V, Prabhudesai S, et al Tissue specific roles for the ribosome biogenesis factor Wdr43 in zebrafish development. PLoS Genet. 2014;10(1):e1004074 Epub 2014/01/30. 10.1371/journal.pgen.1004074 ; PubMed Central PMCID: PMCPMC3907300. 24497835 PMC3907300

[42] Moayedi Y, Basch ML, Pacheco NL, Gao SS, Wang R, Harrison W, et al The candidate splicing factor Sfswap regulates growth and patterning of inner ear sensory organs. PLoS Genet. 2014;10(1):e1004055 Epub 2014/01/02. 10.1371/journal.pgen.1004055 ; PubMed Central PMCID: PMCPMC3879212. 24391519 PMC3879212

[43] Weiske J, Huber O. The histidine triad protein Hint1 interacts with Pontin and Reptin and inhibits TCF-beta-catenin-mediated transcription. J Cell Sci. 2005;118(Pt 14):3117–29. 10.1242/jcs.02437 . 16014379

[44] McBurney MW, Jones-Villeneuve EM, Edwards MK, Anderson PJ. Control of muscle and neuronal differentiation in a cultured embryonal carcinoma cell line. Nature. 1982;299(5879):165–7. . 7110336 10.1038/299165a0

[45] Orsolits B, Borsy A, Madarász E, Mészáros Z, Kőhidi T, Markó K, et al Retinoid machinery in distinct neural stem cell populations with different retinoid responsiveness. Stem Cells Dev. 2013;22(20):2777–93. Epub 2013/07/24. 10.1089/scd.2012.0422 ; PubMed Central PMCID: PMCPMC3787404. 23734950 PMC3787404

[46] Kahn M. Symmetric division versus asymmetric division: a tale of two coactivators. Future Med Chem. 2011;3(14):1745–63. 10.4155/fmc.11.126 . 22004083

[47] Qu D, Hage A, Don-Carolis K, Huang E, Joselin A, Safarpour F, et al BAG2 Gene-mediated Regulation of PINK1 Protein Is Critical for Mitochondrial Translocation of PARKIN and Neuronal Survival. J Biol Chem. 2015;290(51):30441–52. Epub 2015/11/04. 10.1074/jbc.M115.677815 ; PubMed Central PMCID: PMCPMC4683266. 26538564 PMC4683266

[48] Bernstein HG, Stricker R, Dobrowolny H, Trübner K, Bogerts B, Reiser G. Histochemical evidence for wide expression of the metalloendopeptidase nardilysin in human brain neurons. Neuroscience. 2007;146(4):1513–23. Epub 2007/04/18. 10.1016/j.neuroscience.2007.02.057 . 17442499

[49] Yamamoto S, Jaiswal M, Charng WL, Gambin T, Karaca E, Mirzaa G, et al A drosophila genetic resource of mutants to study mechanisms underlying human genetic diseases. Cell. 2014;159(1):200–14. 10.1016/j.cell.2014.09.002 ; PubMed Central PMCID: PMCPMC4298142. 25259927 PMC4298142

[50] Yoon WH, Sandoval H, Nagarkar-Jaiswal S, Jaiswal M, Yamamoto S, Haelterman NA, et al Loss of Nardilysin, a Mitochondrial Co-chaperone for α-Ketoglutarate Dehydrogenase, Promotes mTORC1 Activation and Neurodegeneration. Neuron. 2017;93(1):115–31. Epub 2016/12/22. 10.1016/j.neuron.2016.11.038 ; PubMed Central PMCID: PMCPMC5242142. 28017472 PMC5242142

[51] Mathieu J, Ruohola-Baker H. Metabolic remodeling during the loss and acquisition of pluripotency. Development. 2017;144(4):541–51. 10.1242/dev.128389 ; PubMed Central PMCID: PMCPMC5312031. 28196802 PMC5312031

[52] Qiu R, Wang X, Davy A, Wu C, Murai K, Zhang H, et al Regulation of neural progenitor cell state by ephrin-B. J Cell Biol. 2008;181(6):973–83. Epub 2008/06/09. 10.1083/jcb.200708091 ; PubMed Central PMCID: PMCPMC2426945. 18541704 PMC2426945

[53] Boitard M, Bocchi R, Egervari K, Petrenko V, Viale B, Gremaud S, et al Wnt signaling regulates multipolar-to-bipolar transition of migrating neurons in the cerebral cortex. Cell Rep. 2015;10(8):1349–61. Epub 2015/02/26. 10.1016/j.celrep.2015.01.061 . 25732825

[54] Yang W, Hong YH, Shen XQ, Frankowski C, Camp HS, Leff T. Regulation of transcription by AMP-activated protein kinase: phosphorylation of p300 blocks its interaction with nuclear receptors. J Biol Chem. 2001;276(42):38341–4. Epub 2001/08/22. 10.1074/jbc.C100316200 . 11518699

[55] Angelov D, Verdel A, An W, Bondarenko V, Hans F, Doyen CM, et al SWI/SNF remodeling and p300-dependent transcription of histone variant H2ABbd nucleosomal arrays. EMBO J. 2004;23(19):3815–24. Epub 2004/09/16. 10.1038/sj.emboj.7600400 ; PubMed Central PMCID: PMCPMC522799. 15372075 PMC522799

[56] Alver BH, Kim KH, Lu P, Wang X, Manchester HE, Wang W, et al The SWI/SNF chromatin remodelling complex is required for maintenance of lineage specific enhancers. Nat Commun. 2017;8:14648 Epub 2017/03/06. 10.1038/ncomms14648 ; PubMed Central PMCID: PMCPMC5343482. 28262751 PMC5343482

[57] Sasaki T, Hwang H, Nguyen C, Kloner RA, Kahn M. The small molecule Wnt signaling modulator ICG-001 improves contractile function in chronically infarcted rat myocardium. PLoS One. 2013;8(9):e75010 Epub 2013/09/12. 10.1371/journal.pone.0075010 ; PubMed Central PMCID: PMCPMC3771968. 24069374 PMC3771968

[58] Sasaki T, Kahn M. Inhibition of β-catenin/p300 interaction proximalizes mouse embryonic lung epithelium. Transl Respir Med. 2014;2:8 Epub 2014/09/11. 10.1186/s40247-014-0008-1 ; PubMed Central PMCID: PMCPMC4229507. 25505699 PMC4229507

