# Supplementary material for: Nuclear receptor/Wnt beta-catenin interactions are regulated via differential CBP/p300 coactivator usage
Source: PLoS One. 2018 Jul 18;13(7):e0200714. doi: 10.1371/journal.pone.0200714 (PMC6051640; doi:10.1371/journal.pone.0200714)
Supplement: S1 Fig — (PDF) [file pone.0200714.s001.pdf]

Supplementary Figure Legends (P19 manuscript)

S1 Fig. p300 editing affects Wnt and retinoic acid signaling interactions. Wnt3a and all-trans retinoic acid (ATRA), individually, induced Stra6 (stimulated by retinoic acid 6) mRNA expression in both wild type (WT) P19 cells [left] and p300 edited P19 cells [right], as assessed by realtime RTPCR. In edited P19 cells [right] which have been transfected with empty/control vector (pcDNA), Wnt3a+ATRA treatment does not show any additive effect on Stra6 mRNA expression. However, wild type p300 expression vector (p300) restores the additive effect (observed in WT P19 cells) of Wnt3a+ATRA on Stra6 mRNA expression in edited P19 cells. n =2, $*p < 0.05$. Vehicle/control data (set to 1) as indicated with a red, dashed horizontal.

S1 Fig

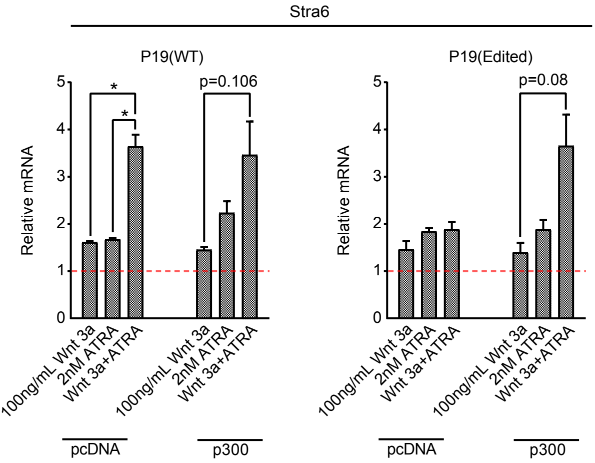

Stra6
P19(WT)
P19(Edited)
Relative mRNA
0
1
2
3
4
5
*
*
p=0.106
p=0.08
100ng/mL Wnt 3a
2nM ATRA
Wnt 3a+ATRA
pcDNA
p300
