# Supplementary material for: Nuclear receptor/Wnt beta-catenin interactions are regulated via differential CBP/p300 coactivator usage
Source: PLoS One. 2018 Jul 18;13(7):e0200714. doi: 10.1371/journal.pone.0200714 (PMC6051640; doi:10.1371/journal.pone.0200714)
Supplement: S1 Table — (PDF) [file pone.0200714.s002.pdf]

S1 Table. 2DICAL Data for IP Experiment

UniProt_ID	protein_description	WT-IG-ip	WT-CBP-ip	WT-p300-ip	Edtd-IG-ip	Edtd-CBP-ip	Edtd-p300-ip	CBPip(Edtd/WT)	p300ip(Edtd/WT)
SMRD1_HUMAN	SWI/SNF-related matrix-associated actin-dependent regulator of chromatin subfamily D member 1 OS=Homo sapiens GN=SMARCD1 PE=1 SV=2null	155.25	310.75	303.8571429	202.75	154.75	229	0.497988737	0.75364363
SMCE1_HUMAN	SWI/SNF-related matrix-associated actin-dependent regulator of chromatin subfamily E member 1 OS=Homo sapiens GN=SMARCE1 PE=1 SV=2null	214.125	258.875	877.125	204.5	205	577	0.791887977	0.657830982
ARI1A_HUMAN	AT-rich interactive domain-containing protein 1A OS=Homo sapiens GN=ARID1A PE=1 SV=3null	249.4166667	257.7692308	404	301.3076923	302	390.4615385	1.17159057	0.966488957
ARID2_HUMAN	AT-rich interactive domain-containing protein 2 OS=Homo sapiens GN=ARID2 PE=1 SV=2	126	104	0	115	71	94	0.682692308	#DIV/0!
SMCA4_HUMAN	Probable global transcription activator SNF2L4 OS=Homo sapiens GN=SMARCA4 PE=1 SV=2null	203	179.25	424.5	303	199.2857143	321.125	1.111775254	0.75647821
BCL7C_HUMAN	B-cell CLL/lymphoma 7 protein family member C OS=Homo sapiens GN=BCL7C PE=1 SV=2null	195.5	136.5	557	262.5	212	513	1.553113553	0.921005386
SNF5_HUMAN	SWI/SNF-related matrix-associated actin-dependent regulator of chromatin subfamily B member 1 OS=Homo sapiens GN=SMARCB1 PE=1 SV=2null	199.5	128.75	1151.75	202.25	298	682	2.314563107	0.592142392
BRD7_HUMAN	Bromodomain-containing protein 7 OS=Homo sapiens GN=BRD7 PE=1 SV=1null	66	91	424	71	72	242	0.791208791	0.570754717
SMRC1_HUMAN	SWI/SNF complex subunit SMARCC1 OS=Homo sapiens GN=SMARCC1 PE=1 SV=3null	181.8571429	182	652	199	233.1428571	490	1.28100471	0.751533742
ROA2_HUMAN	Heterogeneous nuclear ribonucleoproteins A2/B1 OS=Homo sapiens GN=HNRNPA2B1 PE=1 SV=2null	438	408.5	639.5	427.75	492.25	538.75	1.20501836	0.842455043
