# Supplementary material for: Nuclear receptor/Wnt beta-catenin interactions are regulated via differential CBP/p300 coactivator usage
Source: PLoS One. 2018 Jul 18;13(7):e0200714. doi: 10.1371/journal.pone.0200714 (PMC6051640; doi:10.1371/journal.pone.0200714)
Supplement: S2 Table — (PDF) [file pone.0200714.s003.pdf]

S2 Table. 2DICAL Data for Whole Cell Experiment

UniProt_ID	protein_description	WT-Ctrl	WT-RA	WT-w3a	WT-wR	Edtd-Ctrl	Edtd-RA	Edtd-w3	Edtd-wR
FABP5_HUMAN	Fatty acid-binding protein, epidermal OS=Homo sapiens GN=FABP5 PE=1 SV=3null	10796	5625	14984	1931	435	415	437	0
AL1A2_HUMAN	Retinal dehydrogenase 2 OS=Homo sapiens GN=ALDH1A2 PE=1 SV=3	1798	1975	1929	2468	2017	747	2088	317
*SMCA1_HUMAN	Probable global transcription activator SNF2L1 OS=Homo sapiens GN=SMARCA1 PE=1 SV=2null					335	340	295	329
WDR43_HUMAN	WD repeat-containing protein 43 OS=Homo sapiens GN=WDR43 PE=1 SV=3null	2565	893	1426	1403	81	149	187	93
SFR2B_HUMAN	Splicing factor, arginine/serine-rich 2B OS=Homo sapiens GN=SFRS2B PE=1 SV=1null	4352.5	2713	4316.5	1104.5	526	550	340.5	151.5
HINT1_HUMAN	Histidine triad nucleotide-binding protein 1 OS=Homo sapiens GN=HINT1 PE=1 SV=2null	667	2358	4132	398	165	228	784	55
BAG2_HUMAN	BAG family molecular chaperone regulator 2 OS=Homo sapiens GN=BAG2 PE=1 SV=1null	1367	3417.5	1167	1254	1197	757.5	771	549
NRDC_HUMAN	Nardilysin OS=Homo sapiens GN=NRD1 PE=1 SV=2null	1449	1488	1441	1239	1155.5	1120.5	792.5	949
**EFNB1_HUMAN	Ephrin-B1 OS=Homo sapiens GN=EFNB1 PE=1 SV=1null	2277	1141	1738	1639				
*:appeared only in Edtd cell **: appeared only in WT cell									
